# Linear monogamy of entanglement in three-qubit systems

**DOI:** 10.1038/srep16745

**Published:** 2015-11-16

**Authors:** Feng Liu, Fei Gao, Qiao-Yan Wen

**Affiliations:** 1 State Key Laboratory of Networking and Switching Technology, Beijing University of Posts and Telecommunications, Beijing, 100876, China; 2 School of Mathematics and Statistics Science, Ludong University, Yantai, 264025, China

## Abstract

For any three-qubit quantum systems *ABC*, Oliveira *et al.* numerically found that both the concurrence and the entanglement of formation (EoF) obey the linear monogamy relations in *pure* states. They also conjectured that the linear monogamy relations can be saturated when the focus qubit *A* is maximally entangled with the joint qubits *BC*. In this work, we prove analytically that both the concurrence and EoF obey linear monogamy relations in an *arbitrary* three-qubit state. Furthermore, we verify that all three-qubit pure states are maximally entangled in the bipartition *A*|*BC* when they saturate the linear monogamy relations. We also study the distribution of the concurrence and EoF. More specifically, when the amount of entanglement between *A* and *B* equals to that of *A* and *C*, we show that the sum of EoF itself saturates the linear monogamy relation, while the sum of the squared EoF is minimum. Different from EoF, the concurrence and the squared concurrence both saturate the linear monogamy relations when the entanglement between *A* and *B* equals to that of *A* and *C*.

Monogamy is a consequence of the no-cloning theorem^1^, and is obeyed by several types of nonclassical correlations, including Bell nonlocality^2^,^3^,^4^, Einstein-Podolsky-Rosen steering^5^,^6^,^7^,^8^, and contextuality^9^,^10^,^11^. It has also been found to be the essential feature allowing for security in quantum key distribution^12^,^13^.

In its original sense^14^, the monogamy relation gives insight into the way that quantum correlation exists across the three qubits, so it is not accessible if only pairs of qubits are considered. It relates a bipartite entanglement measure *E* between bipartitions as follows:


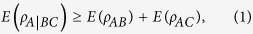


where *A*, *B* and *C* are the respective particles of a tripartite state *ρ*_*ABC*_, each pair *ρ*_*Ai*_ denotes the reduced state of the focus particle *A* and the particle *i* = {*B*, *C*}, and the vertical bar is the notation for the bipartite partition. The original monogamy inequality has been generalized to *n*-qubit systems for the squared concurrence by Osborne and Verstraete^15^. The squared entanglement of formation (SEoF) is also a monogamous entanglement measure for qubits which has been proved by Bai *et al.*^16^,^17^. However, the concurrence and the entanglement of formation (EoF) themselves do not satisfy the monogamy relation. Therefore, it is usually said that the concurrence and EoF are not monogamous. Here, EoF in a two-qubit state *ρ*_*AB*_ is defined as^1^:


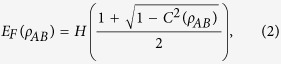


where 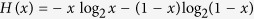 is the binary Shannon entropy and 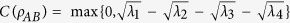 is the concurrence with the decreasing nonnegative *λ*_*i*_ being the eigenvalues of the matrix 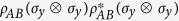.

Recently, Oliveira *et al.*^18^ claimed that violating Eq. (1) does not mean that the concurrence and EoF can be freely shared. In fact, they numerically found that both the concurrence and EoF are linearly monogamous in three-qubit pure states, which means that either of the two entanglement measures satisfies the following inequality


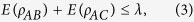


where the upper bound *λ* < 2 is a constant. They conjectured that *λ* = 1.2018 for EoF and the linear monogamy relations can be saturated only when the focus qubit *A* is maximally entangled with the joint qubits *BC*.

Based on the above numerical results, it is natural to ask whether the concurrence and EoF obey the linear monogamy relation for an *arbitrary* three-qubit (pure or mixed) state, whether there exist three-qubit states which saturate these upper bounds, and whether they must be maximally entangled states between the focus qubit *A* and the joint qubits *BC*. In this paper, we prove analytically that both the concurrence and EoF are linearly monogamous in three-qubit states. We also find that the upper bound for *E*_*F*_(*ρ*_*AB*_) + *E*_*F*_(*ρ*_*AC*_) can be attained when two entangled pairs *E*_*F*_(*ρ*_*AB*_) and *E*_*F*_(*ρ*_*AC*_) are equal, while in the same case 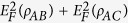 is minimum. Moreover, we verify that the three-qubit pure states must be maximally entangled between qubit *A* and the joint qubits *BC* when they saturate the linear monogamy relation. For the concurrence, we prove analytically that *C*(*ρ*_*AB*_) + *C*(*ρ*_*AC*_) and *C*^2^(*ρ*_*AB*_) + *C*^2^(*ρ*_*AC*_) are maximum when *C*(*ρ*_*AB*_) = *C*(*ρ*_*AC*_). Here, *E*_*F*_(*ρ*_*AB*_) is EoF of a two-qubit state *ρ*_*AB*_, and *C*(*ρ*_*AB*_) is the concurrence between the qubits *A* and *B*.

## Results

This section is organized as follows. In the first subsection, we give a brief review on the linear monogamy conjectures from Oliveira *et al.*^18^ in detail. In the other subsections, we prove exactly that both the concurrence and EoF are linearly monogamous, verify that maximally entangled three-qubit states saturating the linear monogamy relations, and study the distribution of the concurrence and EoF in three-qubit states.

### The linear monogamy conjecture from Oliveira *et al*

The original monogamy relation^14^ gives much insight on the manner in which entanglement is shared across three parties. Then it can be used to characterize genuine tripartite entanglement^17^. However, the linear monogamy relation can only be used to indicate the restrictions for entanglement distribution between *AB* and *AC*. Nonetheless, the linear monogamy relations show that there exist upper bounds on the sum of the two entangled pairs, *E*(*ρ*_*AB*_) + *E*(*ρ*_*AC*_), for the concurrence and EoF themselves, and then the two entanglement measures cannot be freely shared.

For EoF itself, Oliveira *et al.* numerically found the upper bound 1.1882 for *E*_*F*_(*ρ*_*AB*_) + *E*_*F*_(*ρ*_*AC*_), which is considerably smaller than 2. The upper bound is obtained by considering a sampling of 10^6^ random pure states for three-qubit systems. Thus they claimed that it is at least misleading to say that EoF can be freely shared. So, they conjectured that EoF obeys the linear monogamy relation in Eq. (3). Furthermore, Oliveira *et al.* studied a three-qubit pure state 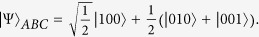 For the state, *E*_*F*_(*ρ*_*AB*_) + *E*_*F*_(*ρ*_*AC*_) ≈ 1.2018 which shows that 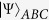 attains the upper bound 1.2018 of the monogamous inequality in Eq. (3) for EoF when the focus qubit *A* is maximally entangled with the joint qubits *BC*. Similarly, they numerically pointed out that the concurrence is linearly monogamous. These numerical results show the squared entanglement measure is different from the entanglement measure itself: the squared concurrence and SEoF both obey the original monogamy relation in Eq. (1), while the concurrence and EoF only obey the linear monogamy relation in Eq. (3). In a word, Oliveira *et al.* found an important phenomenon in the study of the limitations for entanglement distribution.

In the following subsections, we will prove these numerical conjectures.

### Linear monogamy of EoF

A key result of this subsection is to prove analytically that EoF obeys a linear monogamy inequality in an arbitrary three-qubit mixed state, i.e.,


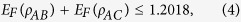


with equality if and only if *E*_*F*_(*ρ*_*AB*_) = *E*_*F*_(*ρ*_*AC*_) = 0.6009.

For proving the general inequality, we first give the following expressions:


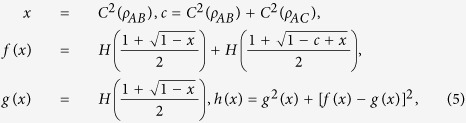


where *x* ∈ [0, *c*], *f*(*x*) = *E*_*F*_(*ρ*_*AB*_) + *E*_*F*_(*ρ*_*AC*_), *g*(*x*) = *E*_*F*_(*ρ*_*AB*_) and 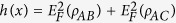. For any three-qubit state *ρ*_*ABC*_, the total amount of entanglement that can be shared is restricted by Eq. (1):


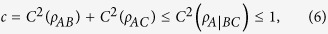


so *c* ∈ [0, 1].

After some deduction, we have


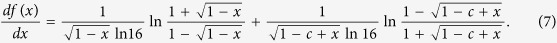


We can deduce that *df*(*x*)/*dx* = 0 when *x* = *c*/2. Therefore, *x* = *c*/2 is a stationary point of *f*(*x*). Finally, we have *d*^2^*f*(*x*)/*dx*^2^ ≤ 0 for any *x* ∈ [0, *c*], and so *f*(*x*) is a concave function of *x*. On the other hand, we have


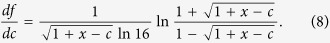


For any *x* ∈ [0, *c*], the first-order derivative is positive. We can deduce that *f* is a monotonically increasing function of *c*. The details for illustrating the above results are presented in **Methods**. Because *x* = *c*/2, i.e., *C*^2^(*ρ*_*AB*_) = *C*^2^(*ρ*_*AC*_), it means *E*_*F*_(*ρ*_*AB*_) = *E*_*F*_(*ρ*_*AC*_) which comes from Eq. (2), and then we have


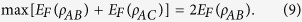


**Figure 1:**
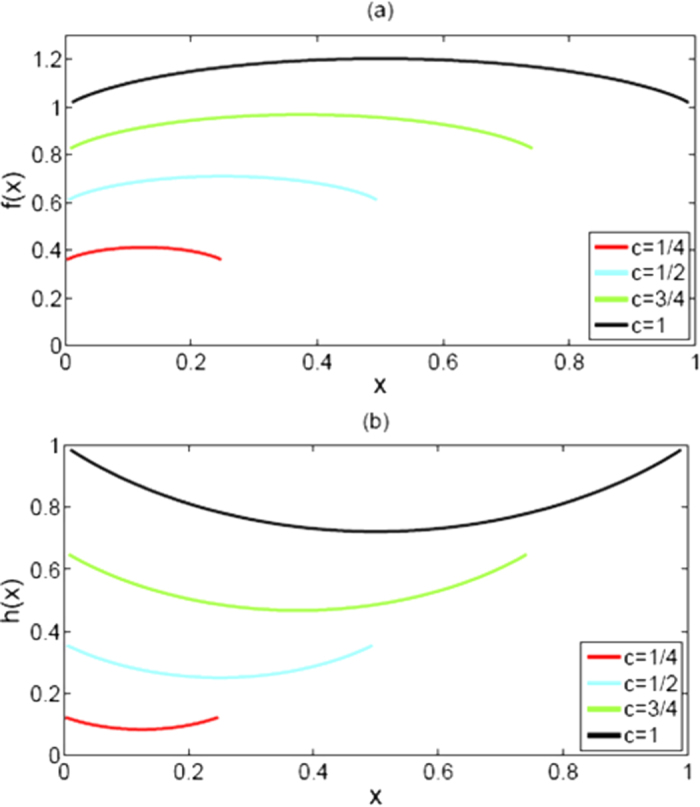
*f*(*x*), the sum of EoF, is a concave function of *x*, while *h*(*x*), the sum of squared EoF, is a convex function of *x*. Their function curves translate upwards as a whole with the growth of *c*.

Finally, we have max *f*(*x*) = *f*(1/2) ≈ 1.2018 and derive the monogamy inequality of Eq. (4), such that we have completed the whole proof showing that EoF is linearly monogamous in three-qubit mixed states. These results can be intuitively observed from Fig. 1(a). Therefore, we draw the conclusion that the conjecture on the linear monogamy from Oliveira *et al.* is true, and the saturation of the upper bound 1.2018 comes from two equal pairs, i.e., *E*_*F*_(*ρ*_*AB*_) = *E*_*F*_(*ρ*_*AC*_).

In the following two paragraphs, we will prove that the conjecture (that the saturated states must be maximally entangled states^19^,^20^ in the bipartition *A*|*BC*) from Oliveira *et al.* is always true in three-qubit pure states using Eq. (4). How to prove the conjecture in three-qubit mixed states is an open problem.

From refs 21,22, we know that any three-qubit pure state 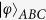 can be written in the generalized Schmidt decomposition


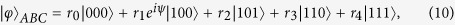


where *ψ* ∈ [0, *π*], *r*_*i*_ ≥ 0, 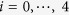 and 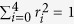. Recently, Zhu and Fei^23^ pointed out that 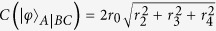, *C*(*ρ*_*AB*_) = 2*r*_0_*r*_2_ and *C*(*ρ*_*AC*_) = 2*r*_0_*r*_3_.

According to Eq. (2) and the result that max [*E*_*F*_(*ρ*_*AB*_) + *E*_*F*_(*ρ*_*AC*_)] = 1.2018 if and only if *E*_*F*_(*ρ*_*AB*_) = *E*_*F*_(*ρ*_*AC*_), we have *r*_2_ = *r*_3_ and 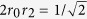. Combining with 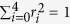, we have 
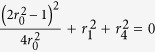
. The equality equals to 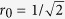 and *r*_1_ = *r*_4_ = 0. Then we have 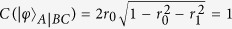. Therefore, 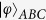 is a maximally entangled state in the bipartition *A*|*BC*, and then the maximum value of *E*_*F*_(*ρ*_*AB*_) + *E*_*F*_(*ρ*_*AC*_) must be attained when the focus qubit *A* is maximally entangled with the subsystem *BC* for three-qubit pure states.

Finally, we study the properties of SEoF, and point out that SEoF is always different from EoF itself.

For discussing the properties of SEoF, we use the expressions in Eq. (5). For any *c* ∈ [0, 1], it is easy to determine that


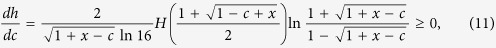


so it is a monotonically increasing function of *c*. After some deduction, we have


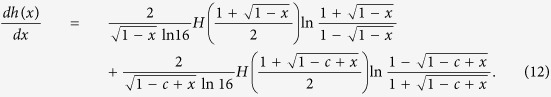


It can be verified that the first-order derivative *dh*(*x*)/*dx* = 0 when *x* = *c*/2. So *x* = *c*/2 is a stationary point of *h*(*x*). The details for illustrating the results have been presented in **Methods**, and they can also be intuitively found out from Fig. 1(b). Moreover, *h*(*x*) is a convex function of *x* from ref. 18, so we have *h*(0) or *h*(*c*) is the maximum value of it. Thus, the saturation of the lower bound for 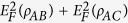 comes exclusively from one of the entangled pairs and there is no state closing to the upper bound with *E*_*F*_(*ρ*_*AB*_) =* E*_*F*_(*ρ*_*AC*_). More specifically, 
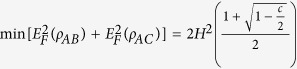
 when *E*_*F*_(*ρ*_*AB*_) = *E*_*F*_*(ρ*_*AC*_). Then, there exist some three-qubit pure states in Eq. (10) satisfying the above specified conditions. From the viewpoint of quantum informational theory, the phenomenon can be interpreted as follows: the more closing to each other of EoF itself in two pairs *AB* and *AC*, the less amount of entanglement in the sum of SEoF.

In the next subsection, we similarly study the linear monogamy of the concurrence and the properties of the concurrence and its squared version.

### Linear monogamy of the concurrence

A key result of this subsection is to prove analytically that the concurrence obeys a linear monogamy inequality in an arbitrary three-qubit mixed state, i.e.,


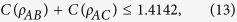


with equality if and only if *C*(*ρ*_*AB*_) = *C*(*ρ*_*AC*_) = 0.7071.

For proving the above inequality, we give the following notations:


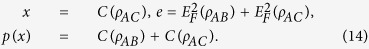


Because SEoF satisfies the monogamy relation for three-qubit states, we find that *e* ∈ [0, 1].

For any *e* ∈ [0, 1], the maximum value of *p* is a monotonically increasing function of *e* if the first-order derivative


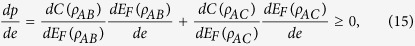


where EoF is a function of the concurrence given by Eq. (2), and *E*_*F*_(*ρ*_*AB*_) and *E*_*F*_(*ρ*_*AC*_) are both implicit functions of *e* given by Eq. (14). *x* = *p*(*x*)/2, i.e., *E*_*F*_(*ρ*_*AB*_) = *E*_*F*_(*ρ*_*AC*_), is a unique stationary point of *p*(*x*) if the first-order derivative


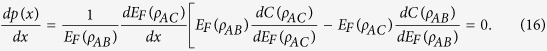


**Figure 2:**
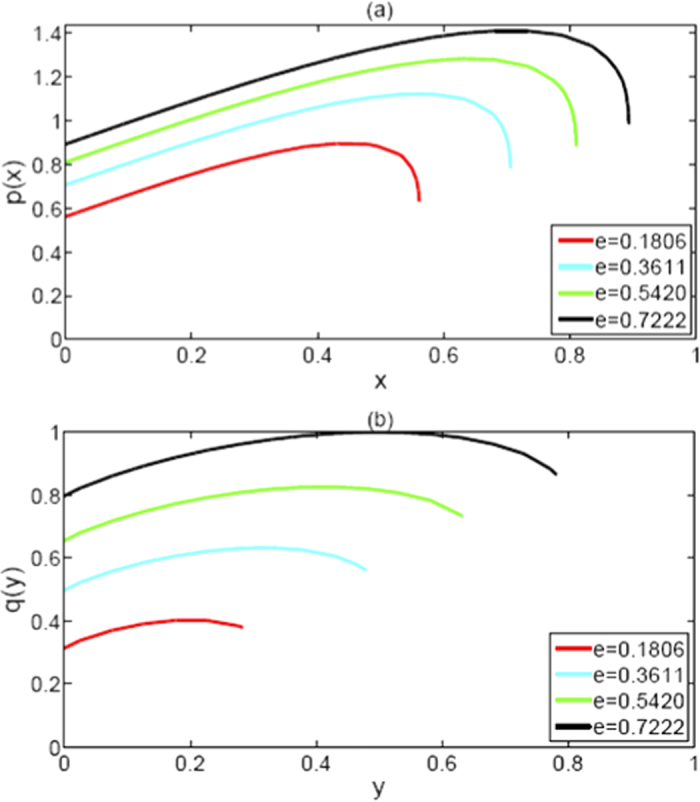
*p*(*x*), the sum of the concurrence, and *q*(*y*), the sum of the squared concurrence, are all concave functions of their own variable. Their function curves translate upwards as a whole with the growth of *e*.

Furthermore, it is not hard to determine that the implicit function *p*(*x*) is a concave function of *x* if the second-order derivative *d*^2^*p*(*x*)/*dx*^2^ ≤ 0. The details for proving the above results are all shown in **Methods**. Then we have max *p*(*x*) = *p*(0.7071) ≈ 1.4142, and derive the monogamy inequality of Eq. (13), such that we have completed the whole proof showing that the concurrence is linearly monogamous in three-qubit mixed states. Here, *x* = *C*(*ρ*_*AB*_) ≈ 0.7071 comes from 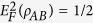 and Eq. (2). These results can be easily verified by a Mathematica program for the binary function *p*, and they can also be intuitively observed from Fig. 2(a). Thus we obtain the conclusion that the saturation of the upper bound 1.4142 also comes from both entangled pairs *AB* and *AC* with equal intensity, i.e., *C*(*ρ*_*AB*_) = *C*(*ρ*_*AC*_).

Moreover, we verify that the conjecture (that the saturated states must being maximally entangled states in the bipartition *A*|*BC*) from Oliveira *et al.* is also ture in general for the concurrence.

Similarly to the example in Eq. (10): According to the relation in Eq. (2) and the result that *C*(*ρ*_*AB*_) + *C*(*ρ*_*AC*_) = 1.4142 if and only if 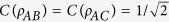, we also obtain *r*_2_ = *r*_3_ and 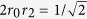. Combining with 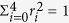, we obtain that 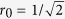 and *r*_1_ = *r*_4_ = 0. Then we have 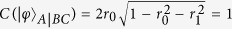. So 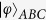 is a maximally entangled state in the bipartition *A*|*BC*, and then the maximum value of *C*(*ρ*_*AB*_) + *C*(*ρ*_*AC*_) can be attained when the focus qubit *A* is maximally entangled with the other two qubits *BC* for any three-qubit pure states.

Finally, we study the properties of the squared concurrence, and point out that the concurrence and its squared version are always similar. The phenomenon is completely different from EoF and SEoF.

Let


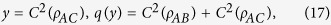


then we have


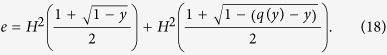


For any *e* ∈ [0, 1], it is not hard to determine that *q*(*y*) is a concave function of *y*, 2*y* is its maximum value, and *y* = *q*(*y*)/2 is a stationary point of *q*(*y*). So the saturation of the upper bound comes similarly from both pairs *AB* and *AC*. More specifically, max[*C*^2^(*ρ*_*AB*_) + *C*^2^(*ρ*_*AC*_)] = 1 if and only if *C*(*ρ*_*AB*_) = *C*(*ρ*_*AC*_) = 0.7071. These results can be proved as the processing of *h*(*x*), and can be intuitively visualized from Fig. 2(b) in a similar way. From the viewpoint of quantum informational theory, the phenomenon can be interpreted as follows: the more closing to each other of the two pairs *C*(*ρ*_*AB*_) and *C*(*ρ*_*AC*_), the more value of entanglement for the concurrence and its squared version exists.

## Discussion and Summary

Different from the original monogamy relation, the linear monogamy relation can only be used to indicate the restrictions for entanglement distribution. In this work, we respectively investigate the linear monogamy relation for the concurrence and EoF. For three-qubit states, we provide analytical proofs that both the concurrence and EoF obey the linear monogamy relations respectively. We also verify that the three-qubit pure states must be maximally entangled between qubit *A* and the joint qubits *BC* when they saturate the linear monogamy relation. Finally, we find there are the following different phenomena in the distribution of the concurrence and EoF: when the entanglement between *A* and *B* equals to that of *A* and *C*, the sum of EoF itself saturates Eq. (4), while the sum of SEoF is minimum. Different from EoF, the sum of the concurrence itself saturates Eq. (13) when the entanglement between *A* and *B* equals to that of *A* and *C*, and the sum of the squared concurrence is maximum at the same condition.

For future work, there are several open questions. Firstly, our results cannot be used to restrict the sharing entanglement in multiqubit states. Then it is interesting to consider whether our method can be modified to facilitate more generalized *n*-qubit states. Secondly, Zhu and Fei^23^ presented the *α*th power monogamy, where the sum of all bipartite *α*th power entanglement may change with different *α*. Therefore, another interesting open question is to study relations between the upper bound of the sum and *α* (particularly for 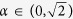). Thirdly, quantum correlations, such as quantum discord^24^,^25^,^26^, generally do not possess the property of the original monogamy relation^27^,^28^,^29^. Our approach may be used to study the linear monogamy properties of quantum correlations.

## Methods

### *c*/2 is a stationary point of *f*(*x*) and *h*(*x*) respectively

In Eq. (5) of the main text, the stationary point of 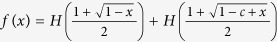 can be obtained if the first-order derivative *df*(*x*)/*dx* = 0. According to Eq. (5), we have


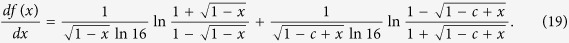


It is easy to verify that 
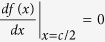
 when *c* ∈ (0, 1), and then *x* = *c*/2 is a stationary point of *E*_*F*_(*ρ*_*AB*_) + *E*_*F*_(*ρ*_*AC*_).

The stationary point of 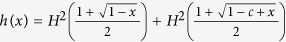 can also be obtained if the first-order derivative *dh*(*x*)/*dx* = 0. According to Eq. (5), we have


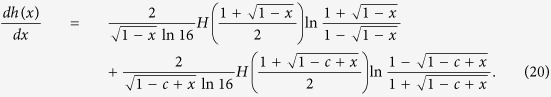


It is easy to verify that 
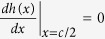
, and then *x* = *c*/2 is a stationary point of 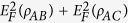.

### *f*(*x*) is concave as a function of the squared concurrence *x*

Let *F*(*x*) = *f*(*x*) − *g*(*x*) with *x* = *C*^2^(*ρ*_*AB*_), and *f*(*x*) and *g*(*x*) being given in Eq. (5) of the main text. This proposition holds if the second-order derivative *d*^2^*f*(*x*)/*dx*^2^ ≤ 0, i.e., *d*^2^*F*(*x*)/*dx*^2^ ≤ 0 and *d*^2^*g*(*x*)/*dx*^2^ ≤ 0. According to the formula of *f*(*x*) and *g*(*x*), we have


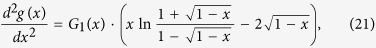


where *G*_1_(*x*) = 1/[8 ln 2 ⋅ *x*(1 − *x*)^3/2^] is a non-negative factor. According to Eq. (7) in ref. 30, we have the second-order derivative *d*^2^*g*(*x*)/*dx*^2^ ≤ 0 in the whole region *x* ∈ [0, *c*].

After some deduction, we have


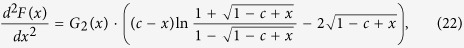


where *G*_1_(*x*) = 1/[8 ln 2 ⋅ (*c* − *x*)(1 − *c* + *x*)^3/2^]. Let *y* = *c* − *x*, then we have *d*^2^*F*(*x*)/*d*(*c* − *x*)^2^ = *d*^2^*g*(*y*)/*dy*^2^, which is non-positive. Therefore, *d*^2^*F*(*x*)/*d*(*c* − *x*)^2^ = *d*^2^*F*(*x*)/*dx*^2^ ≤ 0.

Finally, we have proved that the second-order derivative *d*^2^*f*(*x*)/*dx*^2^ ≤ 0 in the whole region *x* ∈ [0, *c*], and *f*(*x*) is a concave function of *x*.

### *f*(*c*) and *h*(*c*) are both monotonically increasing functions of *c* for any *x*

The monotonically increasing property of 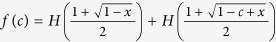 is satisfied if the first-order derivative *df*(*c*)/*dc* > 0. According to Eq. (5), we have


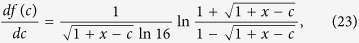


which is positive because 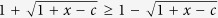 for any *x* ∈ (0, 1). We can deduce that *f*(*c*) is a monotonically increasing function of *c*.

The function 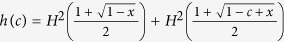 also satisfies the monotonically increasing property if the first-order derivative *dh*(*c*)/*dc* > 0. According to Eq. (5), we have


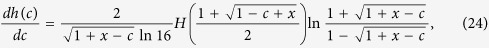


which is positive because 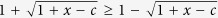 for any *x* ∈ (0, 1). We can deduce that *h*(*c*) is also a monotonically increasing function of *c*.

### The maximum value of *p*(*x*) is a monotonically increasing function of *e*

According to Eq. (2), we know *C*(*ρ*_*AB*_) is a implicit function of *E*_*F*_(*ρ*_*AB*_), and then we have


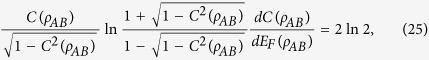


Therefore, *dC*(*ρ*_*AB*_)/*dE*_*F*_(*ρ*_*AB*_) ≥ 0.

From Eq. (14) of the main text, *E*_*F*_(*ρ*_*AB*_) and *E*_*F*_(*ρ*_*AC*_) are both implicit functions of *e*, then we have


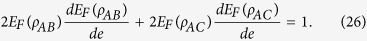


Because *E*_*F*_(*ρ*_*AC*_) is a constant for any *x* ∈ [0, 1], we have *dE*_*F*_(*ρ*_*AC*_)/*de* = 0. Combining with Eq. (26), we know *dE*_*F*_(*ρ*_*AB*_)/*de* > 0.

According to Eq. (14) and the chain rule, we have


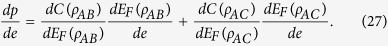


Therefore, *dp*/*de* ≥ 0 and then it is a monotonically increasing function of *e*.

### The derivative functions of *p*(*x*)

According to Eqs. (2) and (14), *E*_*F*_(*ρ*_*AC*_) has the form 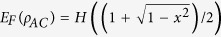. Its first-order derivative is


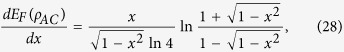


which is positive since the term in the logarithm is larger than 1.

Combining with 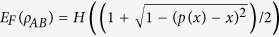, we can get


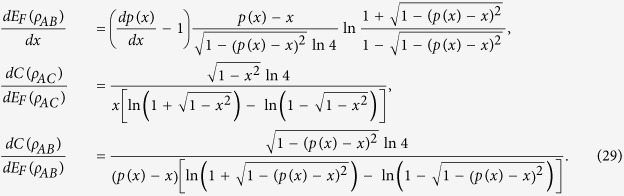


From Eq. (14) of the main text, the first-order derivative has the form


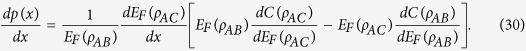


It is easy to verify that *x* = *p*(*x*)/2 is a stationary point of *p*(*x*).

In order to determine the sign of *d*^2^*p*(*x*)/*dx*^2^, we further analyze Eq. (14). After some deduction, we find the second-order derivative of *p*(*x*) satisfies


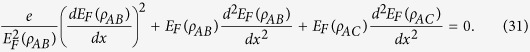


From Eq. (9) in ref. 30, we find that the second-order derivative *d*^2^*E*_*F*_(*ρ*_*AC*_)/*dx*^2^ ≥ 0 and similarly *d*^2^*E*_*F*_(*ρ*_*AB*_)/*dp*^2^(*x*) ≥ 0 in the region *x* ∈ [0, 1]. So the second-order derivative *d*^2^*E*_*F*_(*ρ*_*AB*_)/*dx*^2^ ≤ 0 in the same region. Combining with the chain rule, the second-order derivative *d*^2^*E*_*F*_(*ρ*_*AB*_)/*dx*^2^ can be written as


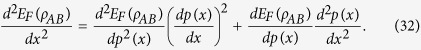


Thus, we prove that the second-order derivative *d*^2^*p*(*x*)/*dx*^2^ ≤ 0 in the whole region *x* ∈ [0, 1], and then complete the proof of the results in the main text.

## Additional Information

**How to cite this article**: Liu, F. *et al.* Linear monogamy of entanglement in three-qubit systems. *Sci. Rep.* **5**, 16745; doi: 10.1038/srep16745 (2015).
